# Data for assigning a proxy variable for office worker in open-ended responses on occupation in Swedish questionnaires

**DOI:** 10.1016/j.dib.2025.112105

**Published:** 2025-09-24

**Authors:** Annika Tillander, Susanna Lehtinen-Jacks, Nisha Singh, Oskar Halling Ullberg, Ulrika Florin, Katarina Bälter

**Affiliations:** a Department of Computer and Information Science (IDA), Division of Statistics and Machine Learning (STIMA), Linköping University, 581 83 Linköping, Sweden; b School of Health, Care and Social Welfare, Division of Public Health Sciences, Mälardalen University, Box 883, 721 23 Västerås, Sweden; c School of Innovation, Design and Engineering, Division of Information Design, Mälardalen University, Box 325, 631 05 Eskilstuna, Sweden; d Department of Medical Epidemiology and Biostatistics, Karolinska Institute, 171 77 Stockholm, Sweden

**Keywords:** Electronic data processing, Epidemiological studies, Occupations, Self report, Surveys and questionnaires, Workplace

## Abstract

In numerous research disciplines, including epidemiology, it is common to compare different occupational categories, such as office workers and non-office workers. When only self-reported occupation titles are available, it is necessary to categorize individuals based on their self-reported titles. Thus, the possibility to identify office workers via self-reported occupation titles can enhance research on the health and well-being of office workers in large population-based epidemiological studies, even without specific questions about office work.

This paper introduces data and R code that can be used to assign a proxy variable for office worker based on responses to an open-ended question (OEQ) about occupation in Swedish questionnaires. The proxy variable is based on the Swedish Standard Classification of Occupations 2012 (SSYK 2012), which includes 8946 occupation titles. Using a translation key, the titles have been categorized into three groups: managers, white-collar workers, and blue-collar workers. White-collar workers (including managers) are considered office workers, while blue-collar workers are classified as non-office workers. The proxy variable has been refined using pilot data from the Swedish population-based epidemiological resource LifeGene.

The R code, together with the proxy variable, can be used in any dataset with a Swedish OEQ about occupation, facilitating the categorization of respondents as either white-collar or blue-collar workers and serving as a proxy variable for office worker. The R code can be used for OEQs regardless of language, provided there is a dataset with a standard classification of occupation in the desired language.

Specifications Table

**Table:** 

Subject	Health and medical sciences / Epidemiology
Specific subject area	Sustainable lifestyle and health among office workers
Type of data	Table, Figure Raw, Analyzed, Filtered
Data collection	The authors wrote R code that can be used to assign a proxy variable for office worker in Swedish questionnaires with responses to an open-ended question (OEQs) about occupation, using R packages stringdist, fuzzyjoin, stringr, and dplyr. The proxy variable is based on the Swedish Standard Classification of Occupations 2012 (SSYK 2012) and a translation key to categorize occupational titles into managers, white-collar workers, and blue-collar workers; both via Statistics Sweden. The Swedish LifeGene pilot data, from the Karolinska Institute, Sweden, was used to refine the proxy variable.
Data source location	Country: Sweden.
	The primary data presented in this paper are stored at Mälardalen University, Västerås, Sweden, and at Linköping University, Linköping, Sweden.
	Secondary data sources used to produce our primary data include: - The Swedish Standard Classification of Occupations 2012 (SSYK 2012) and the translation key for categorizing the SSYK 2012 codes into managers, white-collar workers, and blue-collar workers, which are publicly available on the Statistics Sweden website (see Data accessibility). - The data in the LifeGene resource are collected in Sweden and stored at the Karolinska Institute, Sweden.
Data accessibility	The raw, filtered, and analyzed data are available at: Repository name: Zenodo Data identification number: https://doi.org/10.5281/zenodo.13848203. Direct URL to data: https://zenodo.org/records/16925263. The data includes the following datasets: - Assigning_office_worker_proxy.R, - Occupation_response.xlsx and Occupation_response.csv (number of records = 700), - SSYK12_modified.xlsx and SSYK12_modified.csv (number of records = 6864).
	Instructions for accessing the secondary data sources used for producing our primary data: - Data from the LifeGene resource [1] can be applied by following the instructions given at: https://ki.se/en/research/research-infrastructure-and-environments/core-facilities-for-research/ki-biobank-core-facility-kibb/apply-for-lifegene-data [2].
	
	- The Swedish Standard Classification of Occupations 2012 (SSYK 2012) is publicly available at: https://www.scb.se/dokumentation/klassifikationer-och-standarder/standard-for-svensk-yrkesklassificering-ssyk/ [3]. The website is in Swedish; however, it includes a link to a SSYK 2012 Excel file in English. An English description of the SSYK 2012 is also available at: https://www.scb.se/contentassets/0c0089cc085a45d49c1dc83923ad933a/in-english-ssyk-2012.pdf [4]. - The translation key for categorizing the SSYK 2012 codes into managers, white-collar workers, and blue-collar workers is publicly available at: https://www.scb.se/hitta-statistik/statistik-efter-amne/arbetsmarknad/utbud-av-arbetskraft/yrkesregistret-med-yrkesstatistik/produktrelaterat/Fordjupad-information/nyckel-arbetare-tjansteman-ssyk-2012-xlsx/ [5]. The file is in Swedish.

## 1 Value of the Data

• These data can be used by other researchers in any dataset with a Swedish open-ended question (OEQ) about occupation to categorize respondents into white or blue-collar workers, and serve as a proxy for office worker. The R code can be used for OEQs, regardless of language, provided there is a dataset with a standard classification of occupation in the desired language.
• These data are valuable because, while studies on office workers are often only based on limited samples among office workers, the present data allows research on office workers to be broadened to include large population-based datasets with a variety of both office workers and non-office workers.
• These data can be used in several fields of research. Outside of epidemiology, sociology of labor or urban studies are examples of potential areas where these data may be applied.
• These data were used to identify office workers in the LifeGene epidemiological resource. This allows for further analyses of office workers’ lifestyle behaviors and health in the LifeGene resource, including self-reported data and objective physical and biological measurements, helping to identify factors that are important targets for health promotion among office workers.

## 2 Background

The rationale for compiling these data is to enhance the usability of large population-based epidemiological data for research on office workers’ health in situations where the data lack a direct indicator for office worker. A large proportion of present-day work is performed in office settings and often involves a lot of sedentary time [6], as well as barriers to healthy eating [7], making the office an important area for health promotion. Results from epidemiological studies are needed and may serve as guidance for future health promotion interventions. However, studies on office workers are usually based on limited samples of office workers, while existing large population-based datasets with comprehensive data from questionnaires, as well as physical and biological measurements, often lack specific questions about office work. When a direct indicator for office worker is unavailable, white-collar workers can serve as a proxy for office workers [8], although this group may also include individuals in non-office occupations, such as those working in healthcare or education.

This paper presents data with R code that can be used to assign a proxy variable for office worker based on open-ended question (OEQ) about occupation in Swedish questionnaires. These data can be used in other datasets with Swedish OEQs about occupation to categorize the respondents into white and blue-collar workers, serving as a proxy variable for office worker.

## 3 Data Description

The dataset titled Assigning_office_worker_proxy (1) is an R file consisting of a code to be run in the free, open-source statistical software R; further details about the dataset can be found in the subsection (1) below.

The datasets titled SSYK12_modified (2) and Occupation_response (3) are available in two file types: .xlsx and .csv. The first row in all files is the header, with the naming of each column (variable). A description and potential values of the variables in the two datasets are given in respective subsections (2) and (3) below. Empty cells/spaces indicate missing values. The .csv file uses a semicolon as separator between columns (variables).

**(1) Dataset Assigning_office_worker_proxy**

This dataset consists of R code that can be used to assign a proxy variable for office worker in responses to an OEQ about occupation in Swedish questionnaires. The code starts with reading in the datasets SSYK12_modified and Occupation_response and preparing the data. The dataset Occupation_response can be replaced with optional data as long as it includes two variables named “ID” and “Occupation_swe” (i.e., occupation title given by respondent).

Once the data containing the two required variables has been loaded, a new dataset is created without altering the original data. This step involves cleaning the data by removing rows with missing responses regarding occupation. Additionally, all letters are converted to lowercase, and spaces and symbols are stripped out.

Next in the code is the assignment of the proxy variable for office worker based on the response to “Occupation_swe”, which results in the addition of new variables. The dataset is then split depending on whether there is a perfect match or not. For the subset with perfect matches, the code includes functionality for reporting how many of the observed “Occupation_swe” responses could be assigned the proxy variable, as well as the distribution of office workers. Finally, there is an option to save the new dataset containing the matched occupations. See Appendix A for pseudo code.

**(2) Dataset SSYK12_modified**

This dataset consists of 6864 occupation titles in Swedish and the proxy variable for office worker (white-collar worker). An overview of all the variables in the dataset, along with a short description and possible outcome values of each variable, can be found in Table 1.

**Table 1:** Overview of the variables in the dataset SSYK12_modified (number of records = 6864).

Name of variable	Description of variable	Outcome
Occupation title	Occupation title in Swedish, modified from SSYK 2012 or extracted from LifeGene Pilot dataset	Text/String
From SSYK12	Whether the modified occupation title comes from SSYK 2012 or from LifeGene Pilot dataset	Yes = SSYK 2012, No = LifeGene Pilot
#SSYK12	Number of times the modified occupation title occurs in SSYK 2012	Integer
Proportion white collar	Proportion of times the modified occupation title is coded as white-collar	Value from 0 to 1
Category	Occupation category	White-collar/Blue-collar/Unverified
Office worker	Proxy where office worker corresponds to White-collar	Yes/No/Unverified

**(3) Dataset Occupation_response**

This dataset is an example of what can be extracted from a Swedish questionnaire with an OEQ about occupation. The dataset consists of 700 records and three variables that are explained in Table 2.

**Table 2:** Overview of the variables in dataset Occupation_response (number of records = 700).

Variable name	Explanation	Outcome
ID	A unique identification number that is needed to be able to merge the data later with full questionnaire	Any format
Occupation_swe	Response in Swedish to OEQ about occupation	Text/String
Occupation_eng	English translation of the response in Swedish in variable Occupation_swe	Text/String

OEQ: Open-ended question.

## 4 Experimental Design, Materials, and Methods

R code, in the dataset Assigning_office_worker_proxy, was written by the authors for assigning a proxy variable for office worker in Swedish questionnaires with an OEQ about occupation, using the R packages stringdist (version 0.9.15) [9], fuzzyjoin (version 0.1.6) [10], stringr (version 1.5.1) [11] and dplyr (version 1.1.4) [12].

The basis for the SSYK12_modified dataset comes from the Swedish Standard Classification of Occupations 2012 (SSYK 2012), provided by Statistics Sweden (Table 3) which, in turn, is based on the International Standard Classification of Occupations 2008 (ISCO–08) [13]. The SSYK12_modified dataset was produced in two stages; to differentiate these stages, the dataset following the first stage is called SSYK_reduced and the final dataset following the second stage is called SSYK12_modified. Only the final SSYK12_modified dataset is published in Zenodo.

**Table 3:** *S*econdary data sources that have been used to produce the primary data presented in this paper*.*

Secondary data source	Use of data
The Swedish Standard Classification of Occupations 2012 (SSYK 2012), *n* = 8946 occupation titles with 4-digit codes [3,4]	Produce modified SSYK 2012 codes, and further the proxy variable (presented in the dataset titled SSYK12_modified), together with the Translation key (see next row)
Translation key for categorizing SSYK 2012 codes into managers, white-collar workers, and blue-collar workers [5]	Categorize the modified SSYK 2012 codes into managers, white-collar workers, and blue-collar workers, or unverified when the categorization was not possible
LifeGene Pilot dataset, *n* = 3738 responses on occupation [1,2]	Refine and implement the proxy variable
LifeGene Full dataset, *n* = 23,513 responses on occupation [1,2]	Implement the proxy variable

**Fig. 1:**
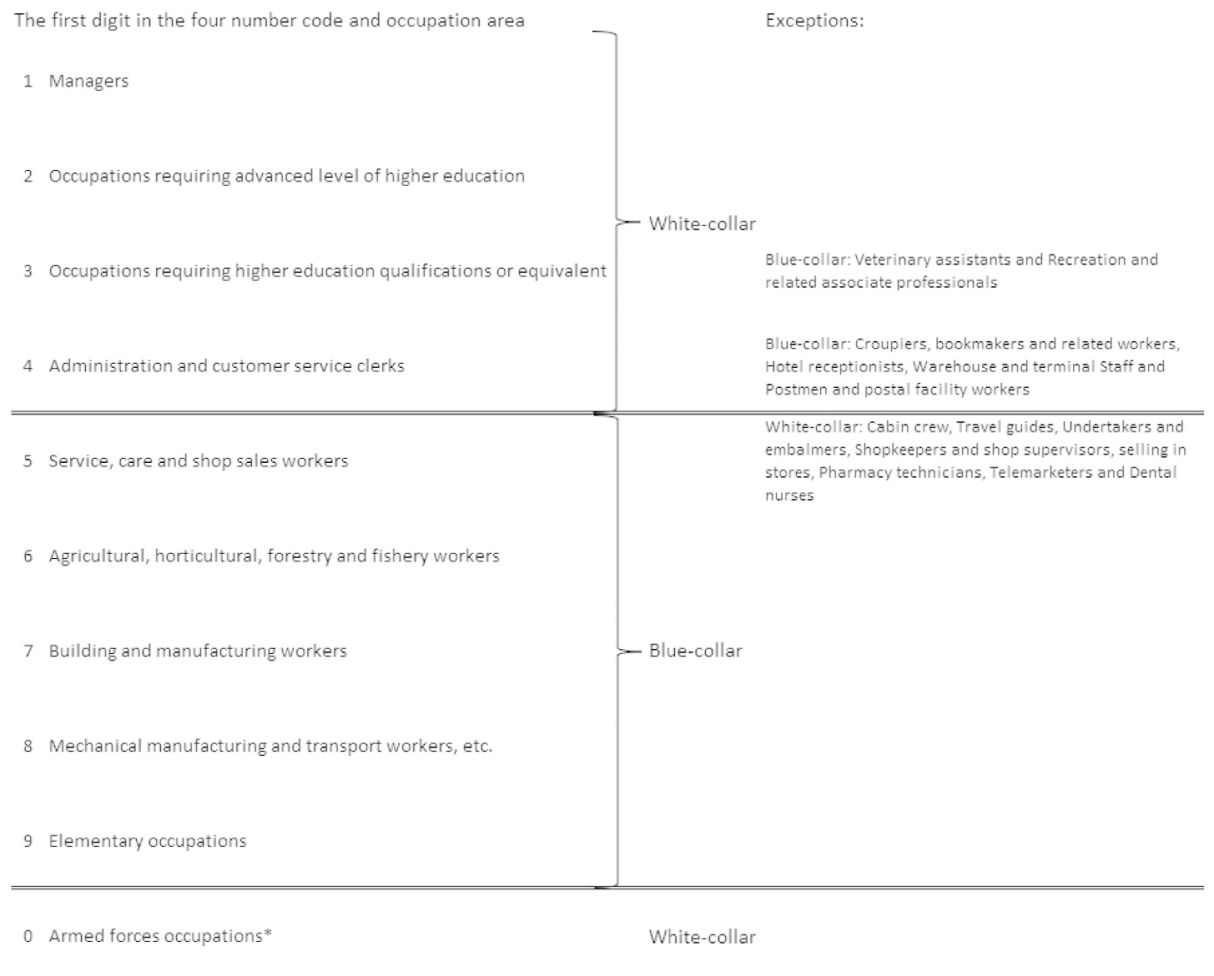
The translation key for suggested categorization based on the four-digit code in SSYK 2012 [3,4]. *Due to the unknown proportion of office work within this occupational group, they were coded as unknown instead of white-collar.

Based on the translation key [5], the 8946 occupation titles in SSYK 2012 can be assigned as white-collar (including managers) or blue-collar workers, as exemplified in Fig. 1.

Armed forces occupation titles, identified by a four-digit code starting with 0, are classified as white-collar workers in SSYK 2012. However, due to the unknown proportion of office work within this occupational group [8], they were coded as unknown instead of white-collar. In addition, 48 occupation titles in SSYK 2012 were assigned the placeholder code “xxxx” due to the absence of a specific four-digit code. For these occupations:

– If a reference to another occupation was provided, the four-digit code of the referred occupation title was used.
– In instances where the referred occupation appeared multiple times with different codes, the first common digits were retained, and the remaining digits were replaced with zeros. When the first digit varied, the majority principle was applied: the most frequently occurring digit was used as the starting digit, and the remaining three digits were set to zero.
– For occupations with the placeholder code and no corresponding occupation with a code, the placeholder “xxxx” was replaced with 10,000 and categorized as unknown in relation to white-collar or blue-collar classification.

To provide a proxy variable for office worker in Swedish questionnaires with an OEQ about occupation, white-collar workers were considered office workers and blue-collar workers were considered non-office workers.

**Fig. 2:**
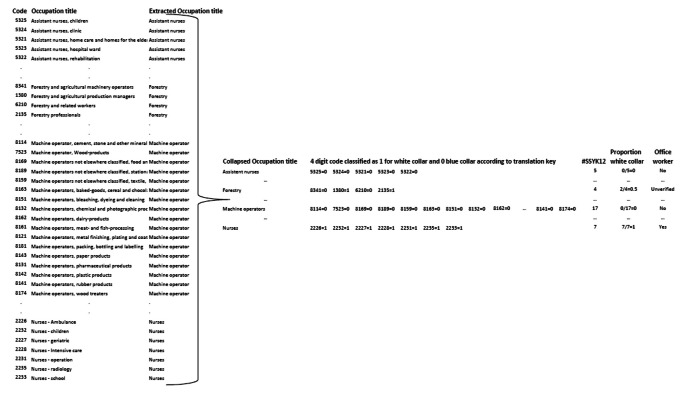
Example of the procedure for extracting occupation titles from SSYK 2012 [3,4] and assigning a proxy variable for office worker based on the four-digit codes in the SSYK 2012.

A challenge was that most respondents did not answer OEQs about occupation with as much detail as required in SSYK 2012, e.g., the four-digit code 2146 in SSYK 2012 corresponds to the occupation title “Engineering professionals in mining-, metallurgy technology and related professionals” but a respondent would most likely answer the OEQ about occupation with “Engineer”. Hence, the first part of the occupation titles in SSYK 2012 was extracted, hereafter referred to as occupation (dataset SSYK12_reduced). Next, the occupations were collapsed into 6601 unique occupation titles and the four-digit codes were assembled, see Fig. 2.

To determine the category of either white-collar, blue-collar, or unknown for each occupation, the majority principle was applied, as follows.

– The frequency of each category was calculated for each occupation title, and the category with the highest frequency was assigned.
– In cases where the frequencies of white collar and blue collar were equal, it was considered a tie, as was the case when all categories had the same frequency.
– If the highest frequency was the same for white-collar and unknown, the category was assigned to white-collar, and similarly for blue-collar.

Next, this information was used to assign a proxy variable for office worker as follows. Given that the occupation title was categorized as white-collar, the occupation was classified as office work. If it was categorized as blue-collar, the occupation was classified as not office work. Finally, if it was categorized as unknown or tied, it was classified as unverified.

To evaluate the proxy variable for office worker, it was compared to the proportion of white-collar workers, calculated as number of white-collar codes for the occupation divided by the number of all codes for a specific occupation title in SSYK 2012 (#SSYK12), see Table 4.

**Table 4:** The distribution of the proxy variable for office worker in relation to the proportion of white-collar among the occupation codes.

Proportion	Office worker		
White-collar*	No	Unverified	Yes
0	2951	41⁎⁎	0
>0 – 0.25	9	0	0
> 0.25 - < 0.5	8	0	0
0.5	0	42	2
> 0.5 – 0.75	0	0	11
> 0.75 - < 1	0	0	12
1	0	0	3525
Sum	2968	83	3550

⁎ Calculated as number of white-collar codes for the occupation divided by the number of all codes for a specific occupation title in SSYK 2012 [3,4].

⁎⁎ Occupation titles without an estimate for the proportion of office work (armed forces occupation titles, *n* = 36), or without a specific four-digit code in the SSYK12 (*n* = 5).

**Fig. 3:**
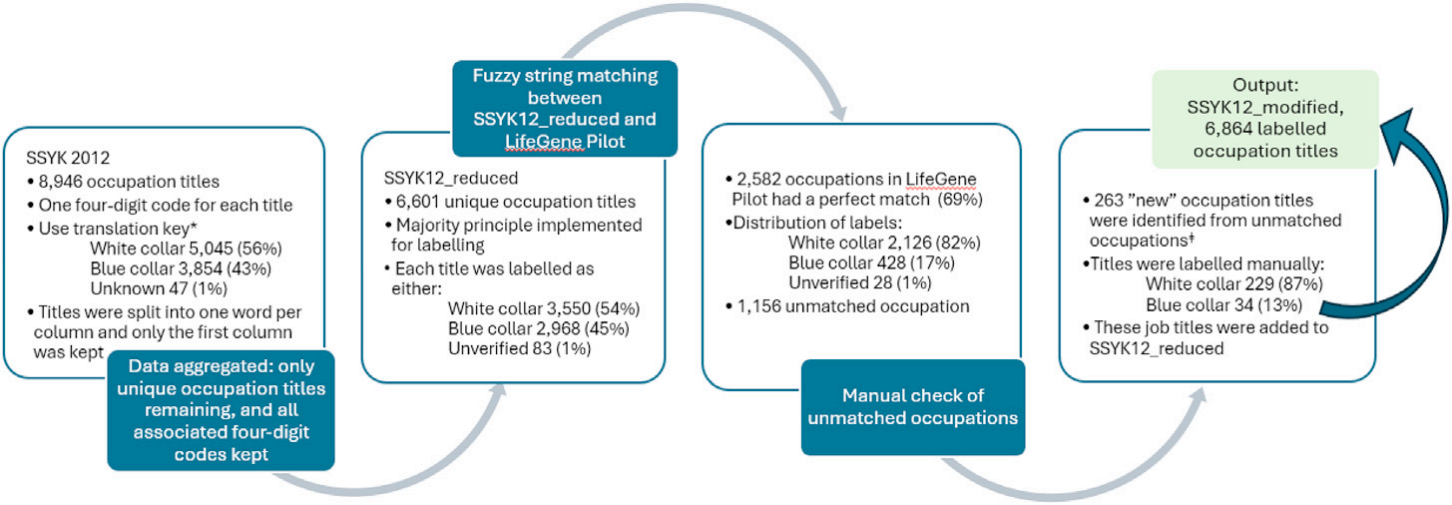
An overview of the process for producing dataset SSYK12_modified. * Given that armed forces occupations have been classified as unknown, and the handling of the placeholder codes “xxxx”. ^ǂ^ Classified as office worker Yes/No according to an evaluation made by two of the co-authors of the present paper.

Next, the SSYK12_reduced dataset was used to assign a proxy variable for office worker in responses given to an OEQ about occupation in a LifeGene Pilot data [1,2]. Fuzzy matching with Jaro distance was used, and 2582 (69 %) out of the 3738 responses given regarding occupation in the LifeGene Pilot dataset had a perfect match (distance = 0) to an occupation in SSYK12_modified. From those 1156 responses in the LifeGene Pilot without a perfect match (distance above 0), a further 263 occupations were identified that could be classified as office worker Yes/No according to an evaluation made by two of the co-authors of the present paper, in full agreement. These occupations were added to the dataset, and renamed at this stage to SSYK12_modified, which finally consists of 6864 occupation titles. An overview of the procedure is presented in Fig. 3**.**

**Fig. 4:**
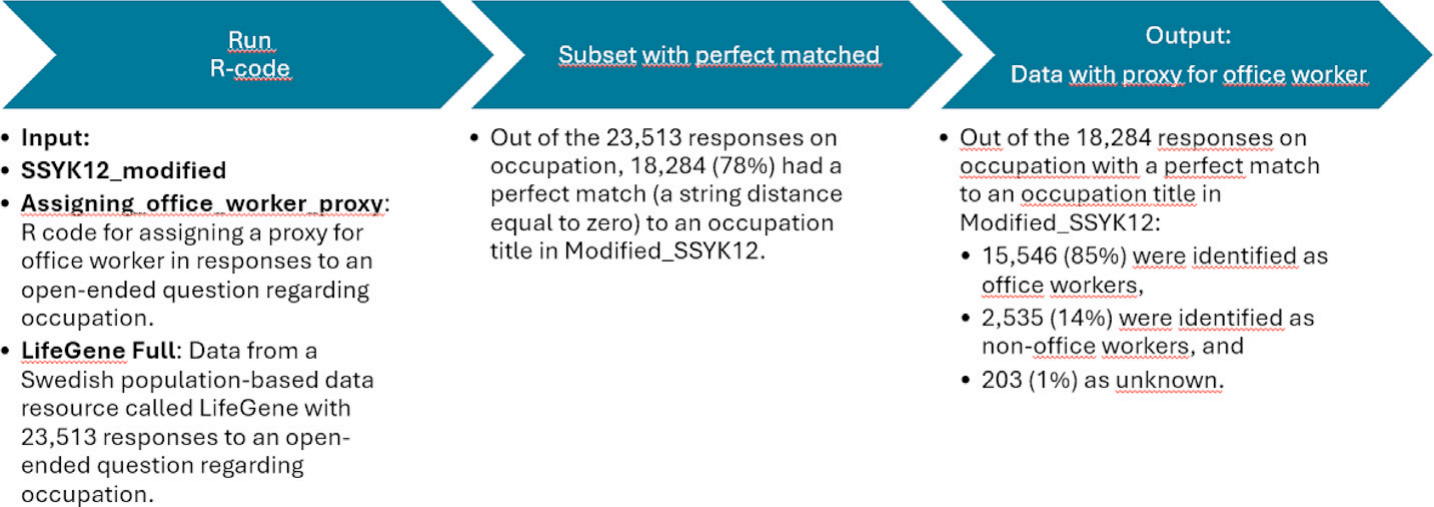
An overview of the process of applying the dataset SSYK12_modified to the LifeGene Full dataset.

The SSYK12_modified dataset was then tested on the main LifeGene data [1,2]. An overview of the process is presented in Fig. 4. Out of the 23,513 responses on occupation in the LifeGene Full dataset, 18,284 (78 %) had a perfect match (zero distance) with the SSYK12_modified dataset. The distributions of occupations following the implementation of the SSYK12_modified dataset on the LifeGene Pilot and LifeGene Full datasets, respectively, are shown in Table 5.

**Table 5:** Implementation of the SSYK12_modified dataset on the LifeGene Pilot and LifeGene Full datasets.

LifeGene dataset	Responses on occupation (N)	Office worker*				
		Yes (N)	No (N)	Unverified (N)	Total (N)	Unmatchedǂ (N)
Pilot	3738	2582	461	28	3071	667
Full	23,513	15,546	2535	203	18,284	5229

⁎ Occupations with a Jaro distance zero (i.e., perfect match) between occupation in SSYK12_modified dataset and occupation in LifeGene dataset were assigned the proxy variable for office worker (Yes/No/Unverified).

ǂ Occupations with Jaro distance above zero (i.e., no perfect match) between occupation in SSYK12_modified file and occupation in LifeGene dataset.

The Occupation_response dataset provides an example of possible responses to an OEQ about occupation, based on the LifeGene pilot data. The self-reported occupations are given in their original format in Swedish (variable Occupation_swe), with a translation to English (variable Occupation_eng) for non-Swedish speaking readers.

Finally, we conducted two comparisons in the LifeGene Full data, as follows. First, we compared the proxy variable office worker with participants’ responses to an OEQ about their workplace to see how many had given “office” as their workplace in the LifeGene Full dataset (Table 6). Many respondents answered by giving the name of the company they worked for. However, we can see that the proportion of respondents who answered “office” was clearly higher among those classified as office workers compared to those respondents who were not classified as office workers or unverified. We also compared the proxy variable office worker with participants’ responses to a validated multiple choice question about their usual level of activity in their daily occupation over the last months [14,15], with five response alternatives ranging from mainly sitting to heavy physical labor (Table 7). The proportion of respondents who answered that their usual activity level was 1 or 2, that is, sedentary, among those classified as office workers, not office workers, and unverified, were 81 %, 23 %, and 44 %, respectively.

**Table 6:** The distribution of the proxy variable for office worker in relation to answering “office” as workplace in the LifeGene Full dataset (*N* = 18,284).

Answered “office” as workplace	Office worker					
	Yes		No		Unverified	
	N	%	N	%	N	%
Yes	9087	58	183	7	50	25
No	6459	42	2352	93	153	75
Total	15,546	100	2535	100	203	100

**Table 7:** The distribution of the proxy variable for office worker in relation to answers about usual level of activity in daily occupation during the last months in the LifeGene Full dataset*.

Usual level of activity in daily occupation during the last months	Office worker					
	Yes		No		Unverified	
	N	%	N	%	N	%
1: Sitting mostly	7951	53	210	9	40	20
2:	4115	28	336	14	47	24
3: Standing and walking mostly	2320	16	1017	42	61	31
4:	469	3	602	25	38	19
5: Heavy physical work	46	0	229	10	13	7
Total	14,901	100	2394	100	199	100

⁎ Due to missing values in Usual level of activity in daily occupation during the last months, *N* = 17,494.

## Limitations

The proxy variable for office worker includes some misclassification for the following reasons: 1) The extent of office work varies between and within occupations and over time. For example, nurses are categorized as white-collar workers and, consequently, are considered office workers [3,4], although one might expect that they perform little office work. However, 92 % of Swedish professionals with a university education in healthcare, including nurses, report engaging in office work for at least 25 % of their working time [8]. 2) The “Unverified” include “Unknown” that refer to occupation titles without an estimate of the proportion of office work in these occupations, and “Tied” that refer to occupation titles that had the same amount of occupation codes referring to white collar (proxy for office work) and non-white collar (non-office work) occupations. For example, all armed forces occupation titles were considered as “Unknown”, even if some of these occupations likely include office work. However, the proportion of “Unverified” within the proxy variable for office worker was not higher than 1 % in the LifeGene Full dataset. 3) Most respondents did not provide detailed answers to OEQs about occupation, making it impossible to apply the translation key with the 4-digit SSYK 2012 codes directly. However, the connection between the reduced and original codes is preserved and can be restored if necessary. 4) For occupation titles with multiple potential 4-digit codes in SSYK 2012 [2,3], we assigned titles to office workers (manager, white-collar workers) or non-office workers (blue-collar workers) based on the majority of specific codes. This approach only considers the number of codes, not the number of people in those occupations. 5) Some responses included words not found in SSYK 2012, such as embassy, own company, bank, leader, and technician. 6) We lack manual coding of the occupation descriptions provided in the LifeGene data, which could be used to assess the percentage of agreement in our semi-automated coding procedure [16].

## Ethics Statement

Informed consent was obtained from participants in the LifeGene project, and the data collection was approved by the Swedish Ethical Review Authority (Etikprövninsgmyndigheten; case number 2009/615-31/1 and case number 2010/1473-31/4). This particular sub-project has also been approved by the Swedish Ethical Review Authority (case number 2021-02549). The research was carried out in accordance with the Declaration of Helsinki.

## Credit Author Statement

**Annika Tillander:** Methodology, Software, Formal analysis, Data Curation, Investigation, Manual categorization of responses to occupation, Validation, Writing - Original draft

**Susanna Lehtinen-Jacks:** Conceptualization, Methodology, Investigation, Manual categorization of responses to occupation, Validation, Writing – Original Draft, Project administration

**Nisha Singh:** Investigation, Writing -Review & Editing

**Oskar Halling Ullberg:** Investigation, Writing -Review & Editing

**Ulrika Florin:** Writing -Review & Editing

**Katarina Bälter:** Conceptualization, Funding acquisition, Supervision, Writing -Review & Editing

## Declaration of Generative AI and AI-Assisted Technologies in the Writing Process

During the preparation of this work, the authors used Microsoft Copilot to assist in generating basic R code. All generated code was manually reviewed, verified, and, where necessary, modified to ensure correctness and appropriateness of the required procedure. After using this tool/service, the authors reviewed and edited the content as needed and take full responsibility for the content in this publication.

## Data Availability

ZenodoData for assigning a proxy variable for office worker in open-ended responses on occupation (Original data)

## Appendix Supplementary materials

Image, application 1
