# Supplementary material for: Data for assigning a proxy variable for office worker in open-ended responses on occupation in Swedish questionnaires
Source: Data Brief. 2025 Sep 24;63:112105. doi: 10.1016/j.dib.2025.112105 (PMC12528912; doi:10.1016/j.dib.2025.112105)
Supplement: Supplementary file 1 [file mmc1.docx]

Appendix A.

Pseudocode Representation of an Algorithm for Assigning an Office Worker Proxy to Open-Ended Occupation Responses

**Input:**

- X: A dataset in .xlsx or .csv format containing at minimum two variables:
 - ID: A unique identifier for each respondent.
 - Occupation_swe: A free-text response to a question regarding occupation, written in Swedish.
- ssyk12_modified: A reference dataset in .xlsx or .csv format containing office worker proxy

**Output:**

- Occupation_ssyk_perfect_match: A subset of responses with exact string matches.
- Occupation_ssyk_distance_above0: A subset of responses matched via fuzzy string similarity with non-zero distance.

**Procedure:**

*1. Data Preparation*: The input dataset X is loaded into a data frame named myData. Next, all rows with missing values (NaN) in the Occupation_swe variable are removed, and the resulting data frame is stored as myData_clean.

2. *Text Normalization:*
 - All characters in Occupation_swe are converted to lowercase.
 - Punctuation marks such as '.', '/', and '-' are replaced with blank spaces ' '.
 - Multiple consecutive spaces (double or triple) are reduced to single spaces.

3. *Fuzzy Matching:* A fuzzy string-matching procedure is applied between myData_clean$Occupation_swe and the occupation labels in ssyk12_modified. The Jaro distance metric is used to quantify string similarity.

4. *Best Match Selection:* For each unique ID, the match with the shortest string distance is retained. This results in a new data frame Occupation_ssyk.

5. *Output Generation:*
 - Responses with a perfect string match (distance = 0) are saved in Occupation_ssyk_perfect_match.
 - Responses with a non-zero string distance are saved in Occupation_ssyk_distance_above0.
